# Supplementary material for: All-polymer piezo-ionic-electric electronics
Source: Nat Commun. 2024 Dec 30;15:10876. doi: 10.1038/s41467-024-55177-y (PMC11686271; doi:10.1038/s41467-024-55177-y)
Supplement: Supplementary file 1 — Supplementary Information [file 41467_2024_55177_MOESM1_ESM.pdf]

Supplementary Information

All-polymer piezo-ionic-electric electronics

Tianpei Xu[1], Long Jin[1, *], Yong Ao[1], Jieling Zhang[1], Yue Sun[1], Shenglong Wang[1], Yuanxiao Qu[1], Longchao Huang[1], Tao Yang[1], Weili Deng[1], Weiqing Yang[1, 2, *]

[1] *Key Laboratory of Advanced Technologies of Materials (Ministry of Education), School of Materials Science and Engineering, Southwest Jiaotong University, Chengdu 610031, People's Republic of China.*

[2] *Research Institute of Frontier Science, Southwest Jiaotong University, Chengdu 610031, People's Republic of China.*

[*] *Corresponding author. E-mail address: longjin@swjtu.edu.cn (Long Jin); wqyang@swjtu.edu.cn (Weiqing Yang).*

Supplementary Note 1

Detailed discussion of the self-polarized bending number experiment

Supplementary Fig. 6a is a diagram of the experimental setup for applying the bending stimulus to PNP devices. We flatten the PNP film device on two acrylic holders. The back and forth motion of the linear motor can apply the bending stimulation to the film at different degrees (curvatures). In order to accurately quantify the degree of bending behavior in terms of curvature, we utilized the film bending model for mechanical analysis, as shown in Supplementary Fig. 6b. The PNP film is tightly centered on the PU encapsulation layer (only one side is shown). When the ends are compressed by a distance ΔL,the bending of the PU will cause the PNP film to bend with the same curvature. Their out-of-plane displacement (h) can be calculated as[1]:

$$h = A\left(1 + cos(2\pi x_1/L)\right)/2 \qquad (1)$$

where A is the bending amplitude and L is the initial length of the film device (The length excluding the fixed portion at each end).

Considering that the PNP film is at the center of the PU and its length is significantly smaller than the PU, the curvature (ω) of the PNP film can be calculated at $x_1 = 0$ (at the center of the device). It is given as:

$$\omega = \left(-4\pi\sqrt{\Delta L/L}\right)/L \qquad (2)$$

Accordingly, the curvature ω of a PNP film at a certain degree of bending can be calculated from the compression distance ΔL.

In order to quantify the bending cycle for bending self-polarization, we fix the bending frequency (1 Hz) and count the number of bending at three typical compression distances (ΔL_1 = 10 mm, ΔL_2 = 20 mm and ΔL_3 = 30 mm). Supplementary Fig. 6c show the actual photographs of the three states. Their curvatures (taking absolute values without considering directionality) increase with the compression distance as $\omega_1 = 0.192\ \mathrm{mm}^{-1}$, $\omega_2 = 0.271\ \mathrm{mm}^{-1}$ and $\omega_3 = 0.332\ \mathrm{mm}^{-1}$, respectively. A group of 20 device-samples under each bending curvature was tested for number of bending. The statistical results are depicted in box-whisker plots and distribution curves, as shown in Supplementary Fig. 6d. The average number of bending for the three curvatures was 278, 125 and 31.75 times, respectively. It is clear that there is a negative correlation between the number of bending and the bending curvature. In fact, greater curvature implies a larger gradient of in-plane strain, which in turn induces a larger piezoionic electric field[2-4]. As a result, the polarization effect is subsequently more pronounced and the number of bends required to achieve the polarized state is decreased. Our experimental data is consistent with this theory. On the other hand, the standard deviation of the number of bending is greater at small curvatures. This is due to the fact that the experimental error becomes larger as the number of bends increases.

Supplementary Note 2

Calculation of β-phase PVDF crystal content within the films.

To begin with, the crystallinity of the film can be obtained by calculating from the DSC test results. From the melting peak area in the DSC curve, the enthalpy of melting of each film can be integrated. Then using the melting enthalpy of 100% crystalline PVDF as a reference, the degree of crystallinity of the film can be calculated, as follows:

$$\chi_C = \frac{\Delta H_m}{w \Delta H_m^0} \times 100\% \qquad (3)$$

Where χ_C is the crystallinity of the sample film, ΔH_m is the melting enthalpy of the sample, w is the mass fraction of PVDF in the sample (For the PNP, the mass of the upper or lower PVDF and the Nafion interlayer is considered to be approximately equal, so w = 2/3) and ΔH_m^0 is standard melting enthalpy of fully PVDF crystals (ΔH_m^0 = 104.5 J g^{-1})[5,6].

Further, the β phase of the PVDF composition in the film relative to the other conformations can be obtained from FTIR spectroscopy. In this work, it is clear that PVDF in PNP and PPP is mainly α- and β-phase, while CP is mainly γ-phase, by combining the data from XRD and FTIR. Therefore, disregarding the γ-phase in PNP and PPP, the relative ratio of the β-phase in them can be calculated by the following equation[7]:

$$F(\beta) = \frac{A_\beta}{\left(\frac{K_\beta}{K_\alpha}\right) A_\alpha + A_\beta} \times 100\% \qquad (4)$$

Where $F(\beta)$ is relative ratio of β-phase in the sample, A_α and A_β are the intensities of the absorption peaks at 764 cm^{-1} and 840 cm-^{1}, K_α and K_β are the absorbance coefficients of above two peaks (K_α= 6.1×10^4 cm^2/mol，K_β=7.7×10^4 cm^2/mol). This formula is based on the fact that FTIR absorption follows the Lambert-Beer law.

Finally, neglecting the effect of the amorphous β-phase, the β-phase crystallinity in the sample (χ_β) can be calculated as:

$$\chi_\beta = \chi_C \times F(\beta) \tag{5}$$

Supplementary Note 3

Detailed description of the short-circuit current phase calibration experiment

As shown in Supplementary Fig. 10a, we connect the signal generator directly to the electrometer, which is equivalent to an AC power supply directly connected to an ammeter. Keep the positive pole of the power connected to the red wire of the ammeter and the negative terminal connected to the black wire, for subsequent experimental control. The signal generator produces a square wave signal with a period of 1s, a duty cycle of 50%, and high and low levels of 1 V and 0 V as an AC source (Supplementary Fig. 10b). The open-circuit current measured by the electrometer was -0.2 mA and 0 mA at high and low levels, respectively (Supplementary Fig. 10c). Thus, it is concluded as follows: when the positive pole of the power is connected to the red wire of the electrometer, the value of the short-circuit current measured is negative.

Based on this important law, the equivalent positive and negative poles of a PNP

device when pressed or released can be accurately determined. Undoubtedly, this helps to further justify bend-self-polarization. Thus, in Supplementary Fig. 10d, when a bending moment in the +Z direction is applied to the PNP, the free protons in the Nafion move toward the top (surface under tension)[8]. The formed piezoionic electric field is from the top to the bottom, which is also the direction of the PVDF dipole after self-polarization. Therefore, when the device is pressurized, its top electrode is equivalently a positive pole and the bottom is a negative pole. Under the same wiring with the calibration circuit, it is supposed to produce a negative current value. The experimental results in Supplementary Fig. 10e are consistent with this speculation, with the device producing a downward short-circuit current when pressed. Thus, it is indeed the piezoionic effect under the bending stimulus that aligns the dipole of the PVDF in Stage I. In the subsequent Stage II, the activated PVDF first makes a piezoelectric response, constraining the piezoionic effect, which together produce an overall electrical signal that conforms to the regularity.

Supplementary Note 4

Molecular dynamics (MD) simulation

MD simulation in this work were performed by Materials Studio 2020 software. A bilayer-polymer interface model was first constructed. It contains 30 PVDF molecular chains (each with a degree of polymerization of 20) and 20 Nafion molecular chains (each containing 60 C atoms in the main chain and 7 long side chains, obtained by random 3:1 copolymerization of two monomers)[9], and the two parts were built together as separate layers. To optimize the conformation and convergence the energy, the model was first geometrically optimized and annealed using the COMPASS II force field of the Forcite module, and then the dynamics were run for 1000 ps (or 2000 ps) under the NVT ensemble and Nose thermostat at a constant pressure and temperature of 298 K.

To reflect the out-of-plane polarization, the change in dipole moment of the PVDF layer during 0-1000 ps was analyzed from the dipole autocorrelation function. In three increasing dynamics time periods, the torsion distributions of the main chain of carbon atoms in the PVDF layer was calculated to obtain the dihedral angle fraction. RDFs between the F atoms of Nafion close to the interface and all H atoms of the PVDF layer were collected at instantaneous states of 1 ps and 1000 ps. In addition, the difference between the two-layer frame energy and the single-layer frame energy sum was calculated to obtain the evolution of the interaction energy in the 0-2000 ps time period, and the corresponding equation is:

$$E_{interr} = E_{bilayer} - \left(E_{layer1} + E_{layer2}\right) \quad (6)$$

Where E_{interr} is two-layer frame total energy, E_{layer1} and E_{layer1} are the energies of the independent PVDF and Nafion layers in the corresponding frames, respectively. All modeling and simulations were performed using Materials Studio 2020 software.

Supplementary Note 5

Direct piezoelectric charge method for testing and calculation d33

At first, in the direct piezoelectric charge measurement method, when subject to out-of-plane pressure, the polarization changes of the sample (P_{sample}) can be calculated as:

$$P_{sample} = \frac{Q_{sample}}{A_1} \tag{7}$$

Where Q_{sample} is the charge transfer between the upper and lower electrodes of the sample, A_1 is the effective area of the sample electrode (the area where the upper and lower electrodes coincide).

The corresponding out-of-plane stress (T_3, subscript "3" indicates the out-of-plane direction) is:

$$T_3 = \frac{F_{sample}}{A_2} \tag{8}$$

Where F_{sample} is the magnitude of the external force on the sample, and A_2 is the area of the force.

In the direct piezoelectric charge measurement method, the direct piezoelectric coefficient d_{33} is calculated by the formula:

$$d_{33} = \frac{P_{sample}}{T_3} \tag{9}$$

When the effective area of the control electrode is equal to the force area, that is, $A_1 = A_2$, the above equation can be simplified as:

$$d_{33} = \frac{Q_{sample}}{F_{sample}} \tag{10}$$

In this work, based on the piezoelectric test system shown in Figure S6, a gradient pressure of 1 to 35 N (F_{sample}) is applied to the sample, and the corresponding charge transfer in the external circuit (Q_{sample}) is measured. Fitting the charge-force function curve, the resulting slope is the d_{33} of the sample[10,11].

In addition, in order to minimize the error, the charge-force test was repeated three times for all samples, and then the average of the three sets was taken as the final result.

Supplementary Note 6

Piezoelectric test methods of contact-separation mode and corresponding triboelectric interference removal strategies

In this work, we employ a contact-separation piezoelectric test mode, as shown in Supplementary Fig. 8 and Supplementary Fig. 21a schematically. In this test platform, linear motor controls the movement of the force gauge probe as well as the acrylic pressing round to achieve the applied force. At the same time, the PNP device (or others) encapsulated with polyurethane on the sample holder is pressed. The resulting transferred charge is measured by the electrometer and is collected.

Possibly, the acrylic round for compression and the encapsulating material polyurethane may generate triboelectric signals due to the contact-separation process[12]. To rule out this possibility, we compared the force loading curve (Supplementary Fig. 21d or e) with the charge curve (Supplementary Fig. 21b) and the voltage curve (Supplementary Fig. 21c) in the same time domain. The results show that there is no phase difference between the force curve and the electrical curve. It is when the acrylic starts to touch the device that the charge curve (or voltage curve) starts to take a plunge (or increase). And when the force disappears, so does the electrical signal. There is no significant signal change during the non-contact period of gradual approach and separation. This force-electric synchronization proves that there is almost no triboelectric signal during the contact separation piezoelectric test.

Considering that we obtained the important parameter of the piezoelectric coefficient d_{33} using the direct piezoelectric charge method (Supplementary Note 4) in the contact-separation mode, we further obtained the accurate transferred piezoelectric charge using the compressed balance analysis (CBA) method proposed recently[12]. The analysis principle is shown in Supplementary Fig. 16. We define the real piezoelectric transfer charge as Q_P. The transferred charge in the Acrylic plate is Q_2, and $-Q_1$ is the transferred charge in the polyurethane encapsulation layer. And q (or q') is the total charge transfer including piezoelectricity and triboelectricity, which is also the charge measured by the electrometer. In Supplementary Fig. 16a, during the compression phase, all charges obey the law of electrostatic balance. Therefore, they have the following relationship:

$$Q_2 - Q_1 + q - Q_P = 0 \quad (11)$$

After that, flip the PNP device so that its positive polarization side is facing out (Supplementary Fig. 16b). The electrostatic balance becomes:

$$Q_2 - Q_1 - q' + Q_P = 0 \quad (12)$$

Combining the above two equations, we have:

$$Q_p = \frac{q + q'}{2} \quad (13)$$

By testing q and q' at different forces, the piezoelectric transfer charge Q_P can be obtained. Therefore, the direct piezoelectric charge method enables the calculation of an accurate d_{33} value that excludes triboelectric interference. Under a gradient force of 1-35 N, the total charge transfer curves of the PNP device with the negative and positive polarization surfaces facing outwards is shown in Supplementary Figs. 16c and d. Sequentially, q and q' were analyzed as in Supplementary Figs. 16e and f to obtain Q_P at each force. And a linear fit was performed to obtain the value of d_{33} under the compressed balance analysis method. The results (Supplementary Fig. 16g) show that the d_{33} obtained by this method is 80.39 pC N^{-1}, which is nearly consistent to the previous method of direct average analysis (80.70 pC N^{-1}, averaging three measurements with only one polarisation surface facing outwards). Also the piezoelectric transfer charges at each force obtained by the two methods are close to each other (Supplementary Fig. 16h). This again demonstrates that the effect of triboelectric signals in the contact-separation piezoelectric test method in this work is almost negligible.

Supplementary Note 7

Calculation of the instantaneous peak power of the PNP

When the PNP (or PPP) operates as a transducer, the instantaneous peak power with external resistance can be calculated according to the DC power calculation formula:

$$P = I^2 \times R \tag{14}$$

Where P is the instantaneous peak power, I is the current, and R is the external resistance.

Supplementary Figures

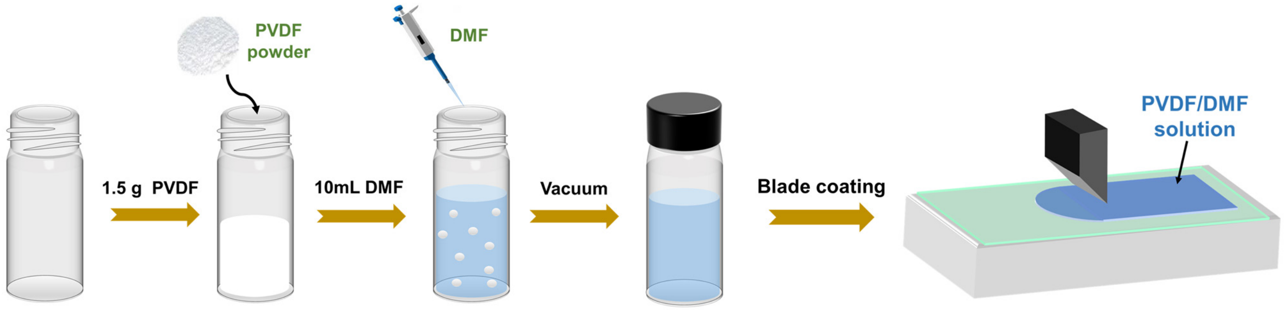

Supplementary Fig. 1 Schematic diagram of the pre-preparation of monolayer PVDF films by blade-coating method.

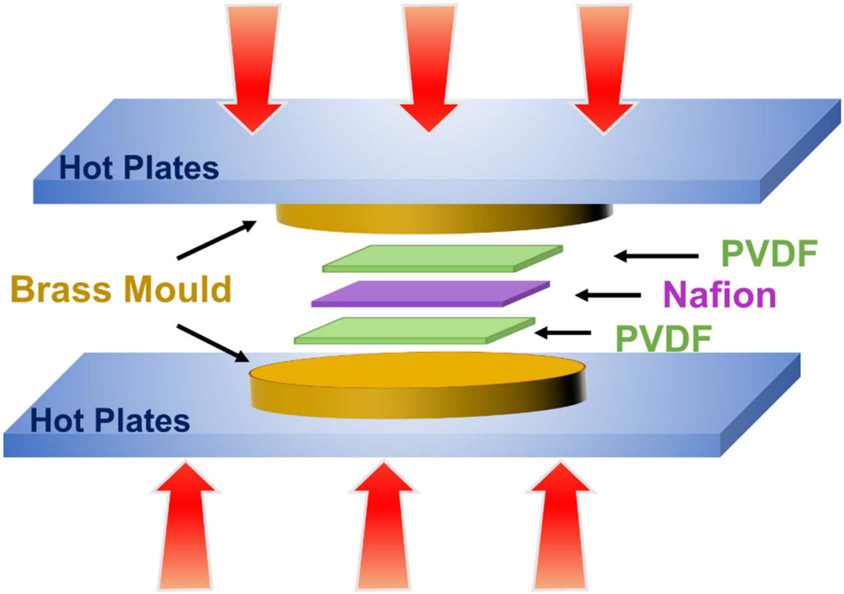

Supplementary Fig. 2 Schematic diagram of a simple laminated hot-pressing method for the preparation of PNP films.

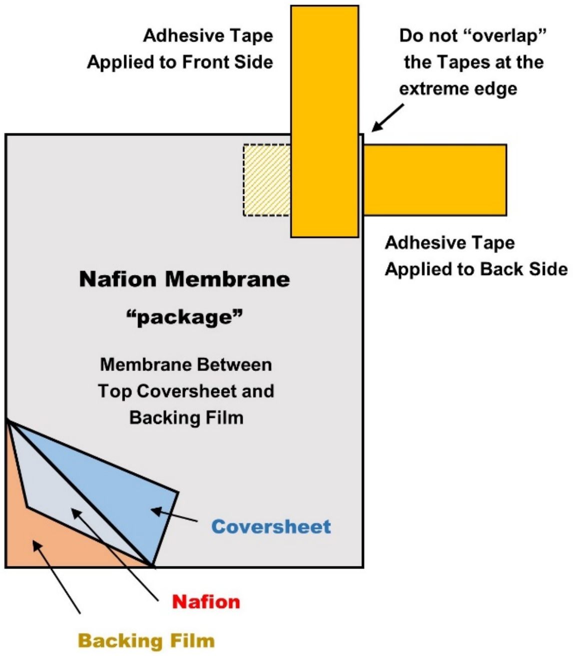

Supplementary Fig. 3 Schematic of Nafion preparation by removing the backing and cover films.

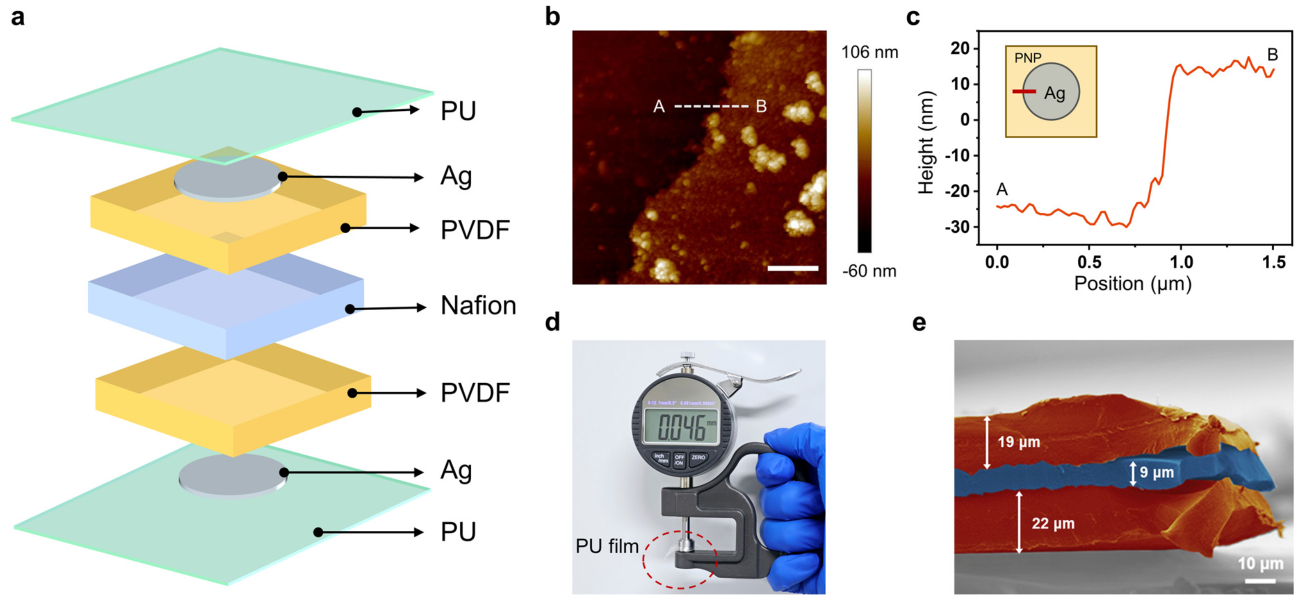

Supplementary Fig. 4 Layer structure of the PNP flexible piezo-ionic-electric device and thickness test of each layer. **a** Schematic representation of the layer components of the PNP device. **b** Atomic force microscope (AFM) morphology result at the step between PNP film and Ag electrode edge. The scale bar is 5 μm. **c** Distribution of surface height along the straight line from position A to position B. The illustration shows the step from PNP film to Ag electrode. **d** Thickness of the encapsulation layer PU form a thickness gauge. **e** Thickness of layers in PNP film obtained from cross-section SEM image. The PVDF layers are orange areas, the Nafion interlayer is blue.

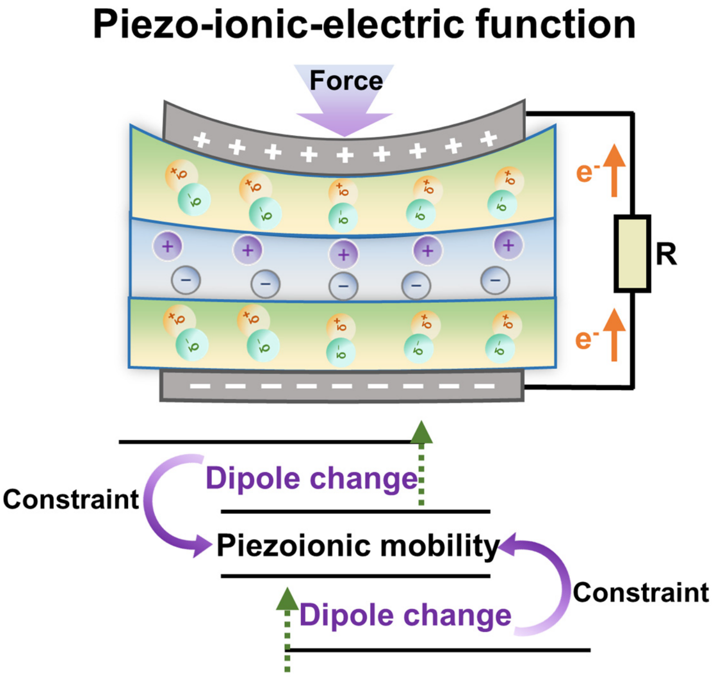

Supplementary Fig. 5 Under vertical stress, piezoelectric electric fields are generated and constrains the piezoionic movement for overall piezo-ionic-electric response.

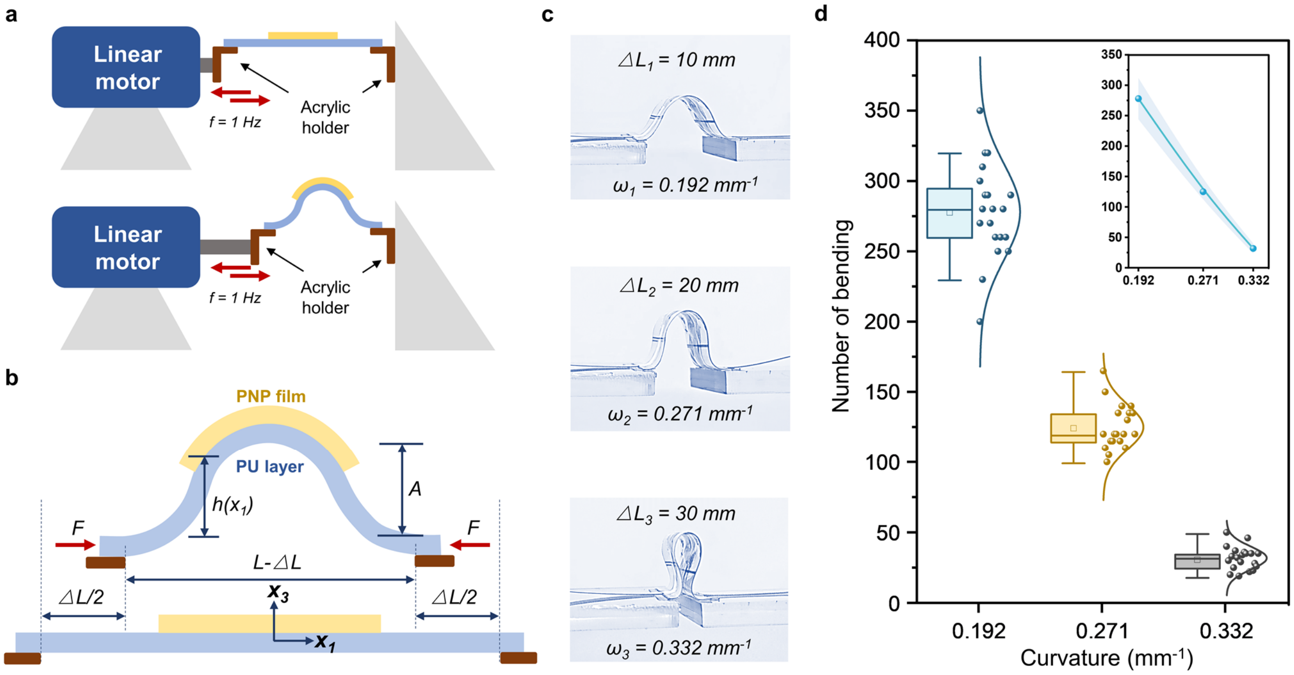

Supplementary Fig. 6 Experiments about bending number for achieving bend-self-polarization. **a** Diagram of the experimental setup for applying the bending stimulus to PNP devices. **b** Bending mechanical model of PNP film-device. **c** Actual photographs of the three typical bending states the corresponding curvatures. **d** Statistical plot of the number of bending required to achieve polarization activation under three curvature conditions, where the statistical distribution of the number of bending is depicted by curves. Boxes, 25-75th percentiles; whiskers, 5-95th percentiles; squares, mean; centre lines, median. Each group has n = 20 independent samples. The inset shows the mean values of the number of bending and their relationship curves under three bending curvatures, and the overlapping part indicates the standard deviation.

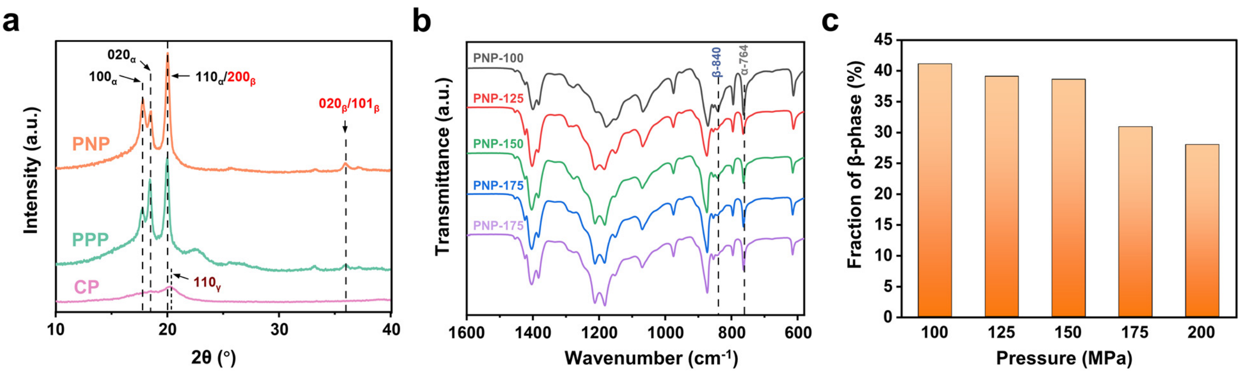

Supplementary Fig. 7 Effect of hot pressing on the condensed state of films. **a** XRD spectra of PNP, PPP and CP. **b** FTIR spectra of PNPs under gradient hot-pressing pressures. **c** The relative content of β-phase PVDF in each PNP calculated from FTIR spectra.

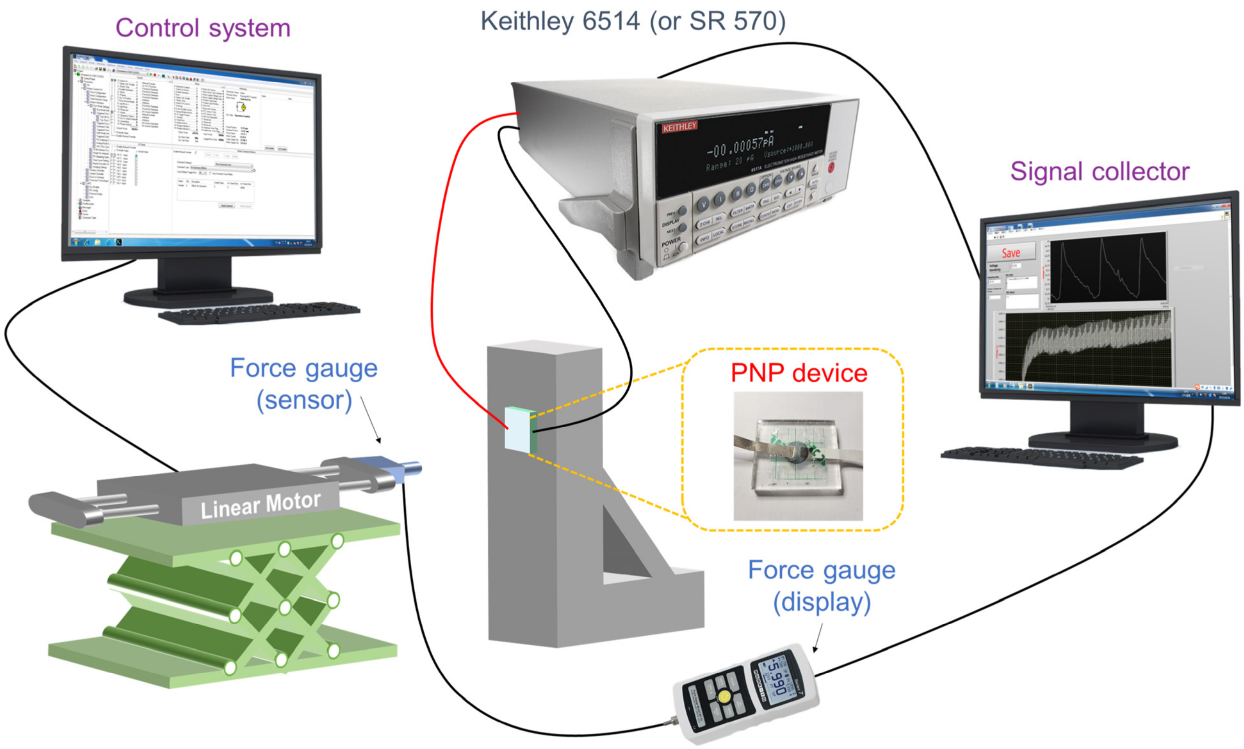

Supplementary Fig. 8 Composition diagram of the piezoelectric signal test system.

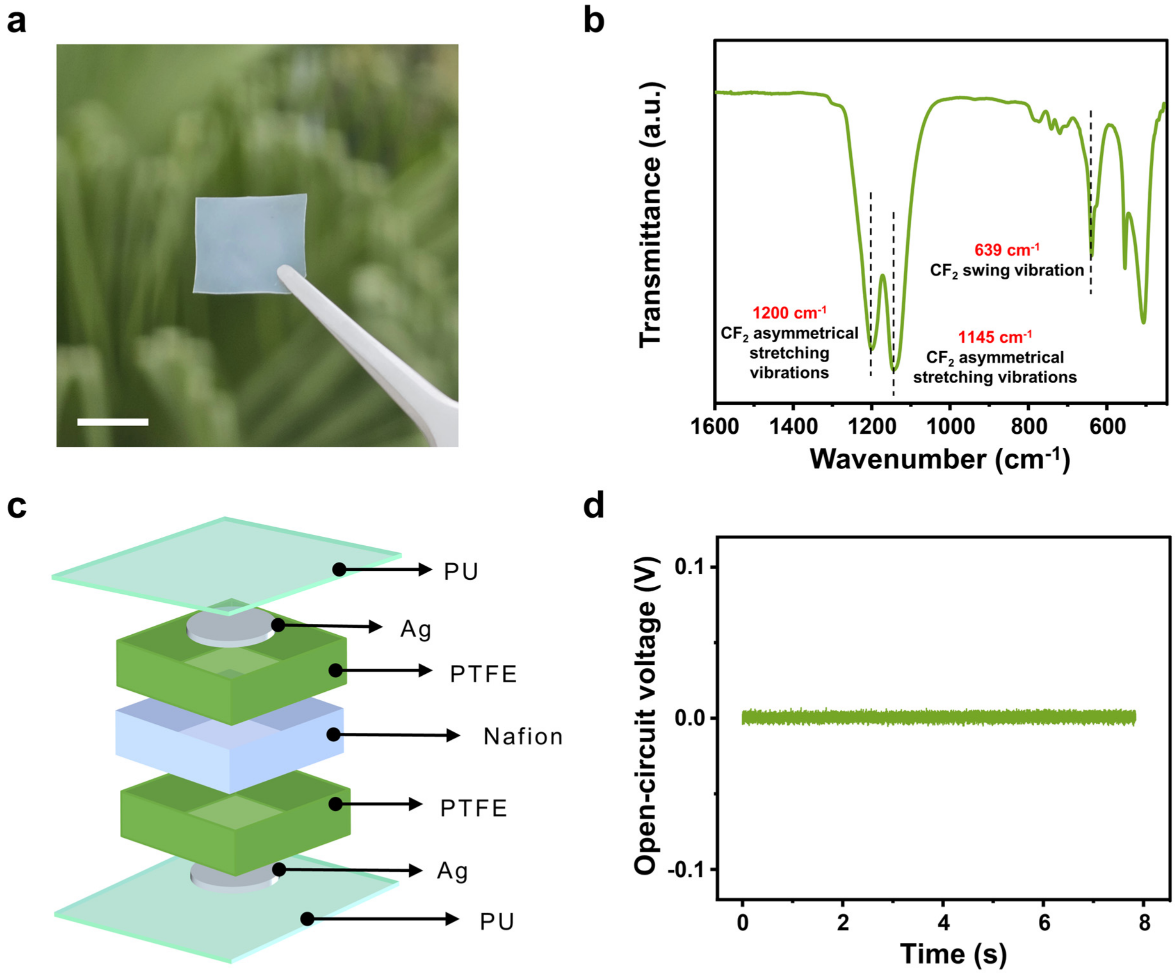

Supplementary Fig. 9 Control experiments on PTFE sandwich films with Nafion interlayers. **a** Photograph of the PTFE-Nafion-PTFE film. The scale bar is 10 mm. **b** FTIR in attenuated total reflectance (ATR) mode for this film. The results show the PTFE characteristic peaks[13]. **c** Structural diagram of the PTFE-based device, for piezoelectric performance testing. **d** The corresponding piezoelectric test data showed no piezoelectric properties of this PTFE-based device.

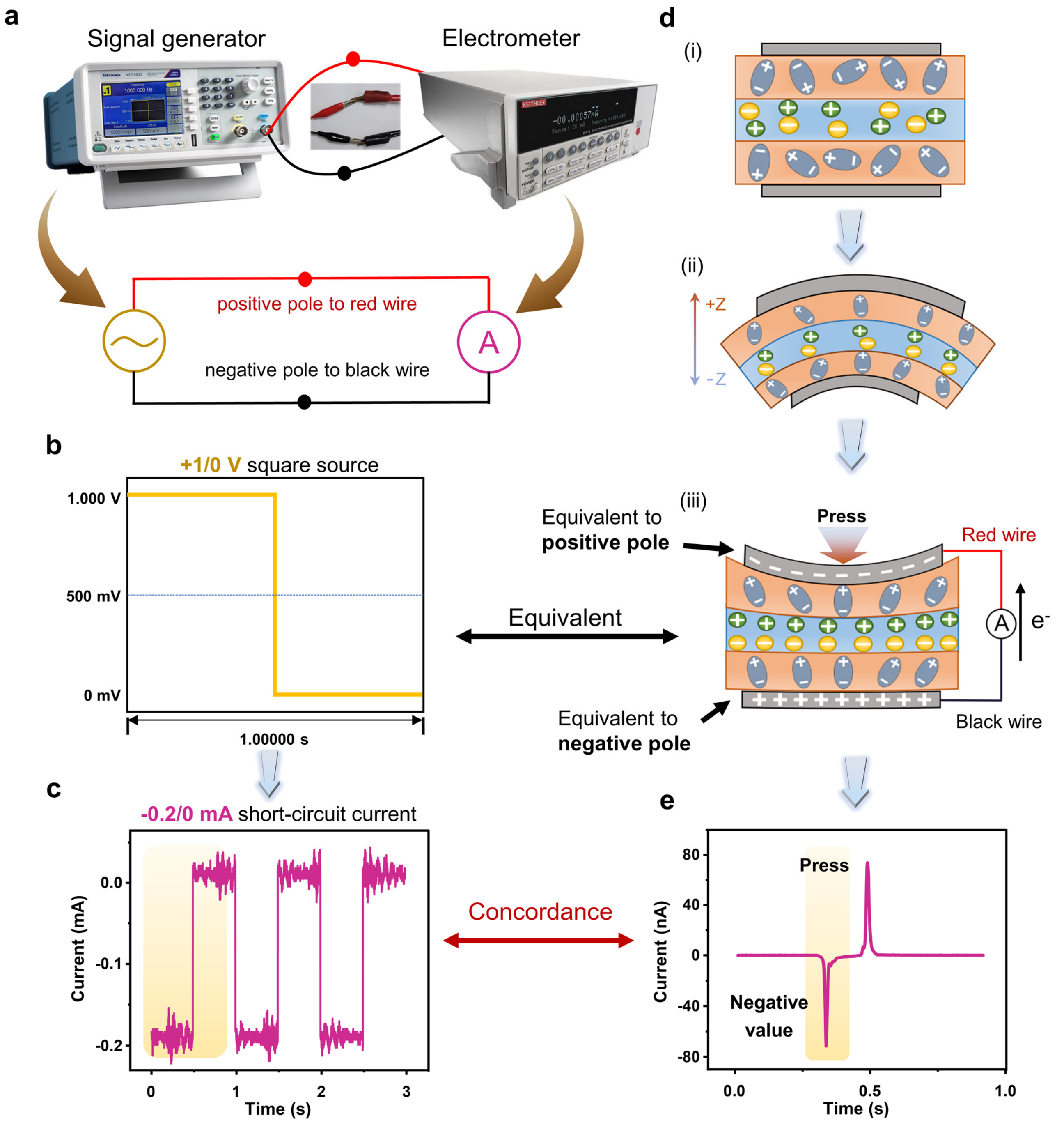

Supplementary Fig. 10 Current phase calibration to assist in verification of bend-self-polarization dominated by piezoionic effect. **a** Electrometer synergized with signal generator for calibrating the short-circuit current phase, and the corresponding equivalent circuit. **b** The signal generator produces a square wave signal as a power source. **c** The square-wave short-circuit current detected by the electrometer is negative

value. **d** After bending the PNP device in the +Z direction, according to the principle of bend-self-polarization, the tensile surface is equivalent to the positive pole of the power supply, and the compressed surface is the negative pole. **e** With the wire connections consistent with calibration circuit, the device produces a negative current when stressed, which is in accordance with the results of the calibration circuit.

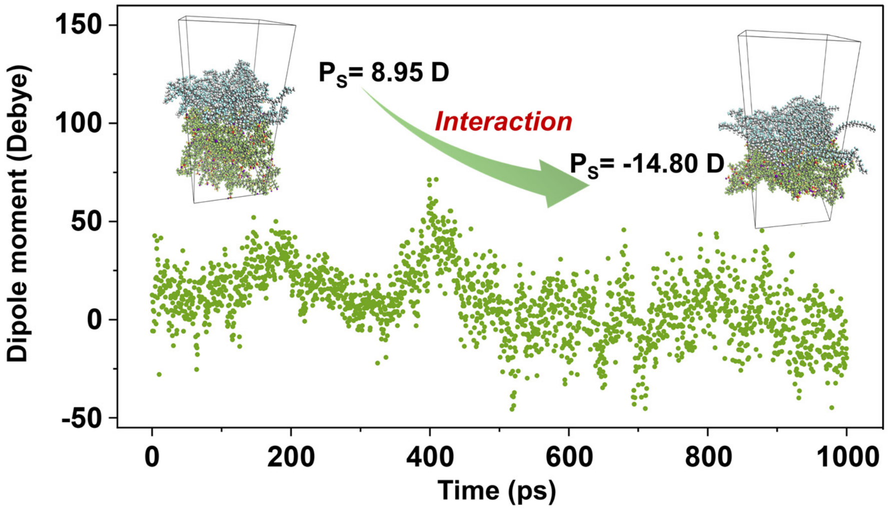

Supplementary Fig. 11 Dipole moments evolution of PVDF layers during 1000 ps molecular dynamics simulations.

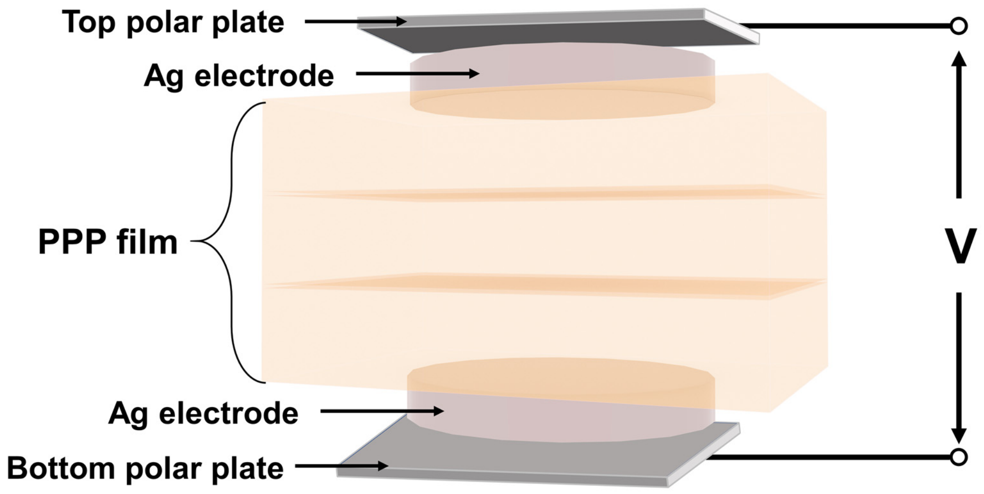

Supplementary Fig. 12 Schematic diagram of electrical-poling for PPP film. The applied voltage was a DC high voltage of 2 kV for 10 min.

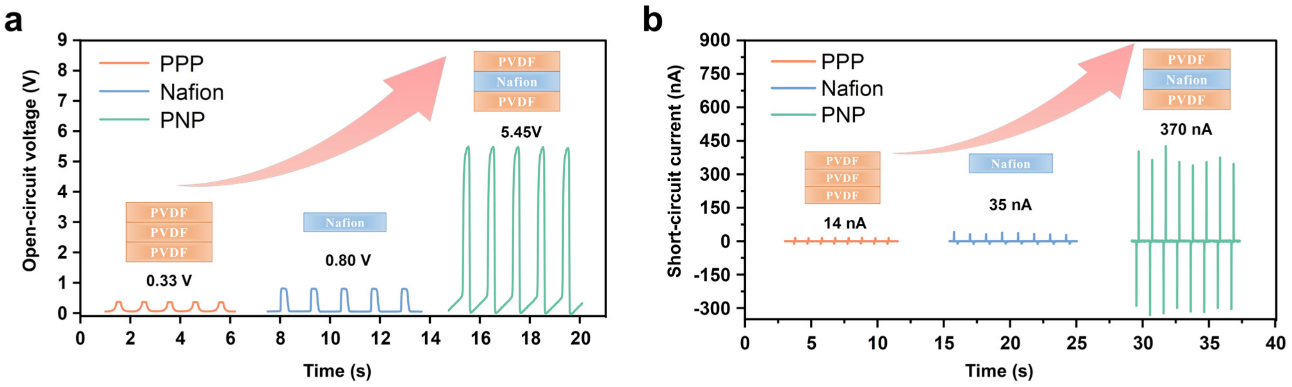

Supplementary Fig. 13 Plots of **a** open-circuit voltage and **b** short-circuit current for PNP against control (PPP and Nafion) under the same test conditions.

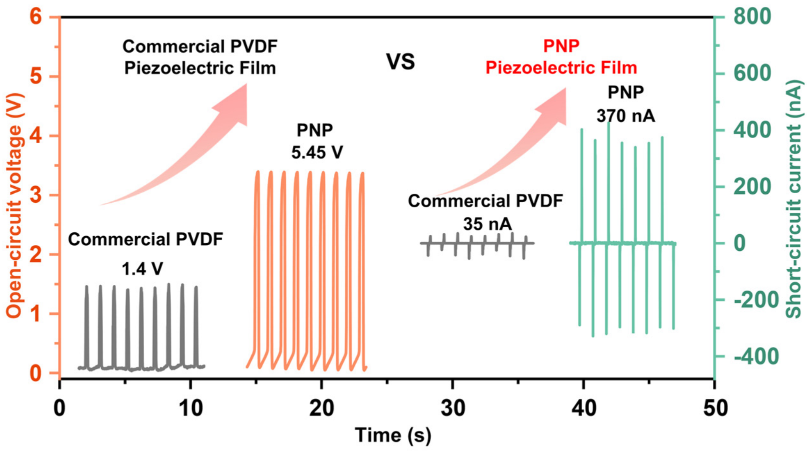

Supplementary Fig. 14 Plot of open-circuit voltage and short-circuit current for PNP against commercial PVDF piezoelectric film under the same test conditions (The commercial film was purchased from PolyK).

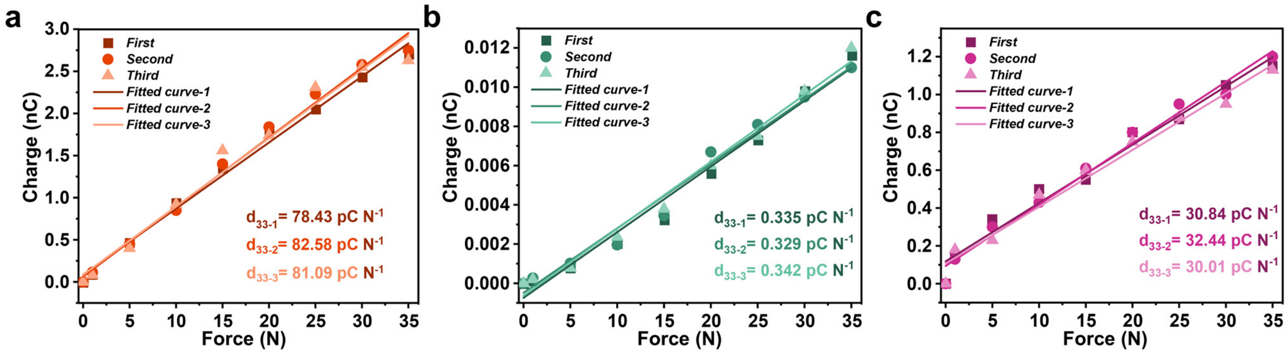

Supplementary Fig. 15 Repeatability experiments for direct piezoelectric charge test of d_{33} of **a** PNP sample, **b** PPP sample and **c** commercial PVDF sample.

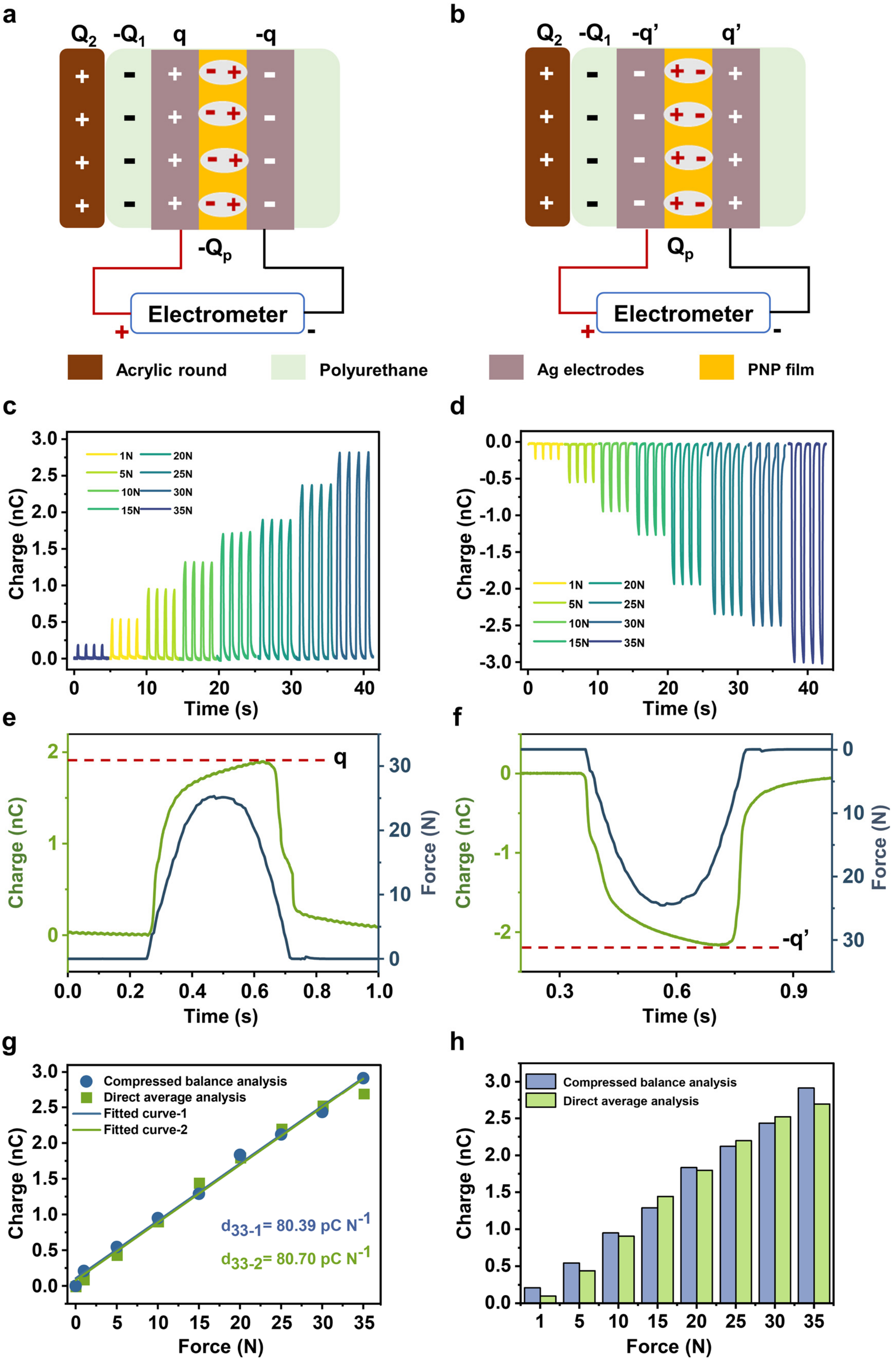
a
b
Q2 -Q1 q -q
Q2 -Q1 -q' q'
-Qp
Qp
Electrometer
Electrometer
Acrylic round
Polyurethane
Ag electrodes
PNP film
c
d
Charge (nC)
Time (s)
1N 5N 10N 15N 20N 25N 30N 35N
e
f
Charge (nC)
Force (N)
Time (s)
q
-q'
g
h
Compressed balance analysis
Direct average analysis
Fitted curve-1
Fitted curve-2
d33-1= 80.39 pC N-1
d33-2= 80.70 pC N-1
Force (N)

Supplementary Fig. 16 Testing real piezoelectric charges by compressed balance analysis method for d_{33} evaluation. Principles of compressed balance analysis, including **a** negative polarization direction and **b** positive polarization direction after flipping. The total charge transfer curves of the PNP device with the **c** negative and **d** positive polarization surfaces facing outwards. The total transferred charge measured from the **e** negative and **f** positive polarization surfaces, respectively, under a force of 25N. **g** Force-charge curves obtained based on the compressed balance analysis method and comparison with existing direct average method. **h** A case-by-case comparison of the piezoelectric transferred charges measured under each force by the two methods.

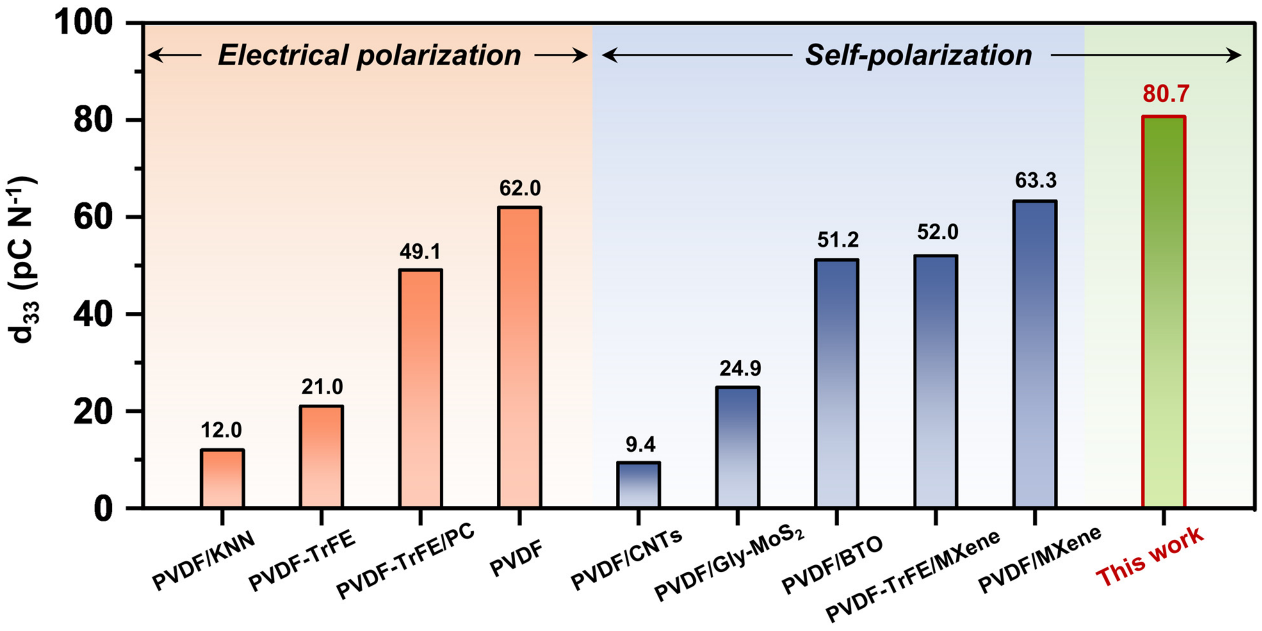

Supplementary Fig. 17 Comparison of d_{33} values between PNP films and recent PVDF-based piezoelectric systems[10,11,14-20].

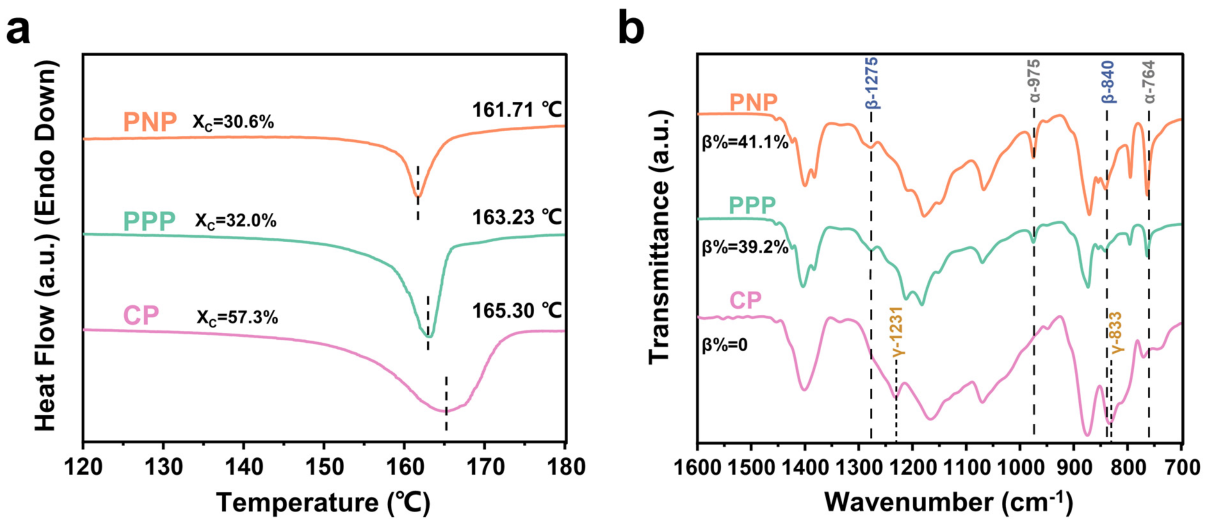

Supplementary Fig. 18 The analysis of β-crystal content aimed at decoupling the variables of piezoelectric influences. **a** DSC curves of PNP, PPP and CP with the corresponding calculated crystallinity. **b** FTIR spectra of PNP, PPP and CP with the corresponding calculated β-phase ratio.

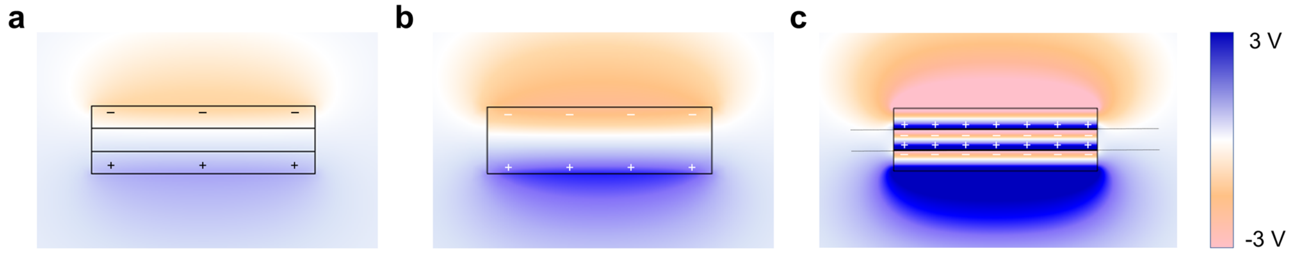

Supplementary Fig. 19 The simulations of space charge distribution of models of **a** PPP, **b** Nafion and **c** PNP by COMSOL Multiphysics 6.0.

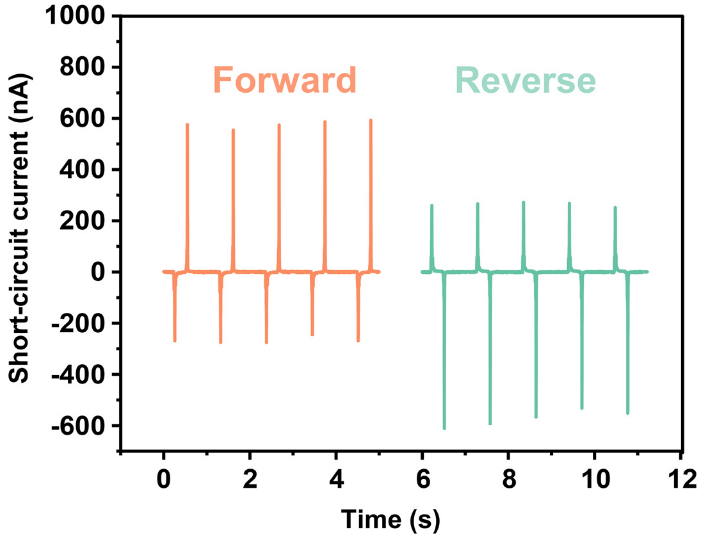

Supplementary Fig. 20 Comparison of the PNP piezo-ionic-electric signals under forward and reverse connection conditions.

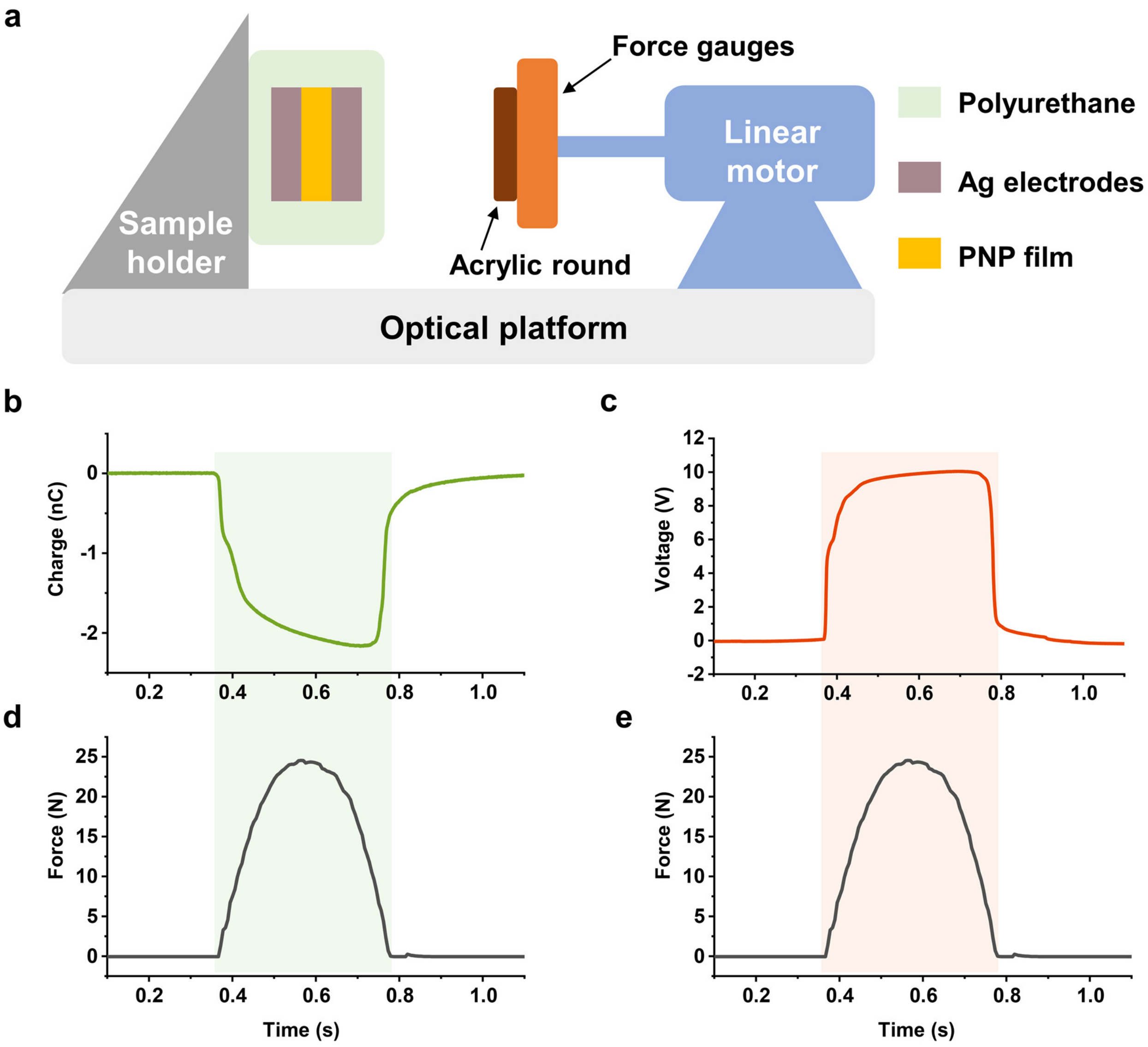

Supplementary Fig. 21 Exclusion of triboelectric signals using force loading curves. **a** Schematic diagram of the contact separation piezoelectric test platform used in this work. **b** Transferred charge curve and **d** corresponding force loading curve. **c** Open circuit voltage curve and **e** corresponding force loading curve.

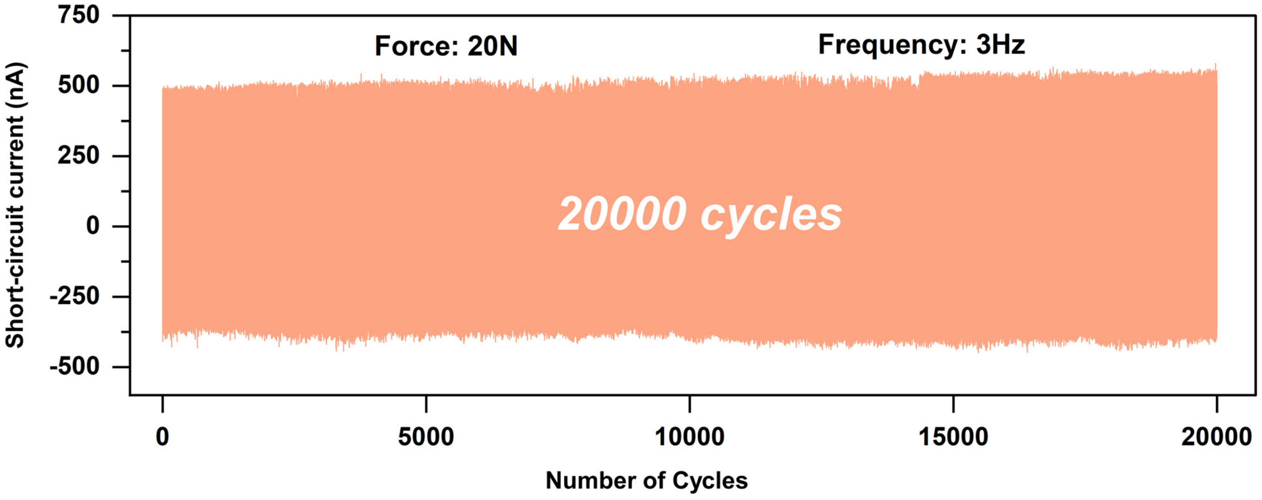

Supplementary Fig. 22 Cyclic stability test results of PNP under 20,000 vertical press excitations.

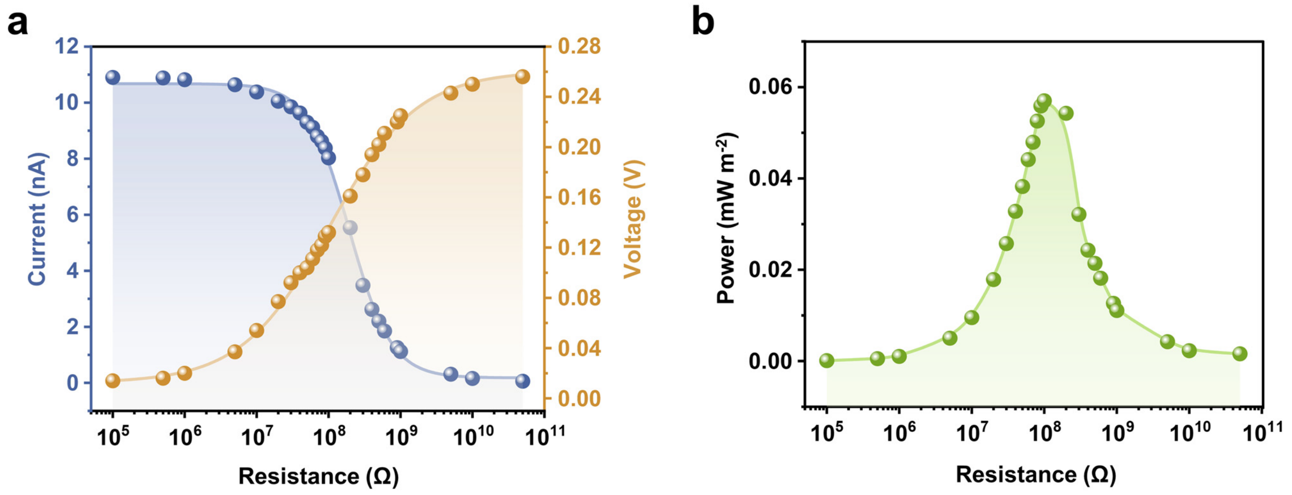

Supplementary Fig. 23 Output test of the PPP with external resistance. **a** Dependence of output current and voltage on external resistance. **b** Instantaneous peak power per unit area with external resistance.

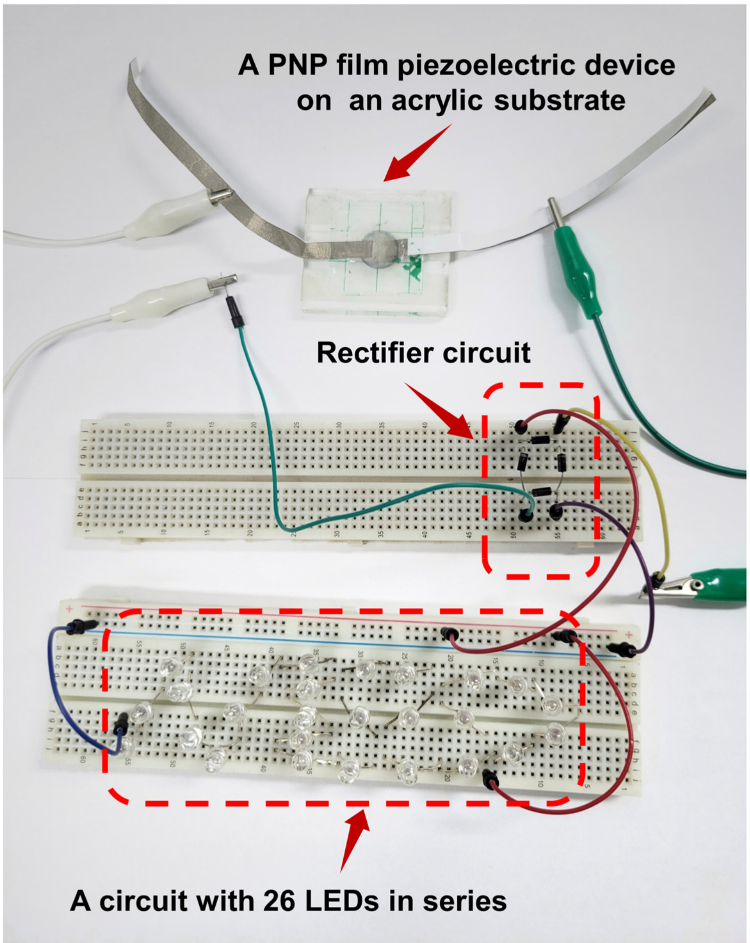

Supplementary Fig. 24 Actual circuit diagram of the PNP film transducer with a rectifier bridge to light LEDs in series.

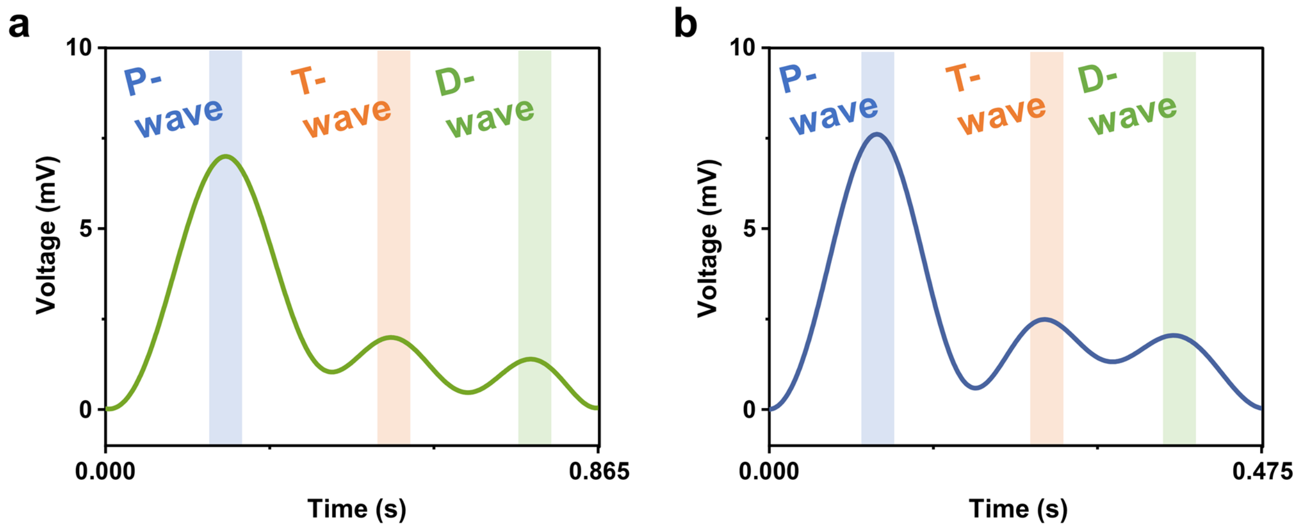

Supplementary Fig. 25 Single-cycle amplification curves of pulse wave signals detected by the PNP. **a** Waveform at rest. **b** Waveform post workout.

Supplementary Table 1

Thickness of constituent layers in PNP thin film devices.

**Layer part**	PU	Ag	PVDF (upper)	Nafion	PVDF (below)
**Thickness**	46 µm	45 nm	19 µm	9 µm	22 µm

Supplementary Table 2

Comparison about pressure sensitivity of PVDF and its copolymer-based piezoelectric films and their corresponding pressure ranges.

Piezoelectric functional material	Pressure range (kPa)	Pressure sensitivity (mV kPa^{-1})	References
PVDF/Nafion	9-221 & 211-354	51.50 & 19.79	Our work
PVDF/ZnO	1.8-451	3.12	21
PVDF-TrFE	20-100	4.56	22
PVDF	21.4-200	7.64	23
PVDF	13.3-88.68	22.6	24
PVDF/$BaTiO_3$	1-40	17	25
PVDF-TrFE	8-75	50	26
PVDF-TrFE/ Graphene Oxide	24-320	13.4	27

Supplementary Table 3

Comparison of d_{33} values between PNP films and recent PVDF-based piezoelectric systems.

Piezoelectric functional material	Self-polarizing or not	d_{33} (pC N^{-1})	References
PVDF/Nafion	Yes	80.7	Our work
PVDF/KNN	No	12	14
PVDF-TrFE	No	21	15
PVDF-TrFE/PC	No	49.1	16
PVDF	No	62	10
PVDF/CNTs	Yes	9.4	17
PVDF/Gly-MoS_2	Yes	24.9	18
PVDF/BTO	Yes	51.2	11
PVDF-TrFE/MXene	Yes	52	19
PVDF/MXene	Yes	63.3	20

Supplementary references

1. Dagdeviren, C. et al. Conformal piezoelectric energy harvesting and storage from motions of the heart, lung, and diaphragm. *Proc. Natl. Acad. Sci. U. S. A.* **111**, 1927-1932 (2014).
2. Li, L. et al. A MXene heterostructure-based piezoionic sensor for wearable sensing applications. *Chem. Eng. J.* **482**, 148988 (2024).
3. Lu, C., Liao, X., Fang, D. & Chen, X. Highly Sensitive Ultrastable Electrochemical Sensor Enabled by Proton-Coupled Electron Transfer. *Nano Lett.* **21**, 5369-5376 (2021).
4. Liu, Y. et al. Self-Powered Piezoionic Strain Sensor toward the Monitoring of Human Activities. *Small* **12**, 5074-5080 (2016).
5. Lanceros-Méndez, S., Mano, J. F., Costa, A. M. & Schmidt, V. H. FTIR AND DSC STUDIES OF MECHANICALLY DEFORMED β-PVDF FILMS. *J. Macromol. Sci., Part B: Phys.* **40**, 517-527 (2001).
6. Teyssedre, G., Bernes, A. & Lacabanne, C. Influence of the crystalline phase on the molecular mobility of PVDF. *J. Polym. Sci., Part B: Polym. Phys.* **31**, 2027-2034 (1993).
7. Gregorio, J., Rinaldo & Cestari, M. Effect of crystallization temperature on the crystalline phase content and morphology of poly (vinylidene fluoride). *J. Polym. Sci., Part B: Polym. Phys.* **32**, 859-870 (1994).
8. Dobashi, Y. et al. Piezoionic mechanoreceptors: Force-induced current generation in hydrogels. *Science* **376**, 502-507 (2022).
9. He, H. et al. Supramolecular Anchoring of Polyoxometalate Amphiphiles into Nafion Nanophases for Enhanced Proton Conduction. *ACS Nano* **16**, 19240-19252 (2022).
10. Huang, Y. et al. Enhanced piezoelectricity from highly polarizable oriented amorphous fractions in biaxially oriented poly(vinylidene fluoride) with pure β crystals. *Nat. Commun.* **12**, 675 (2021).
11. Huang, Z. X. et al. Self-poled piezoelectric polymer composites via melt-state energy implantation. *Nat. Commun.* **15**, 819 (2024).
12. Chen, C. et al. A method for quantitatively separating the piezoelectric component from the as-received "Piezoelectric" signal. *Nat. Commun.* **13**, 1391 (2022).
13. Piwowarczyk, J. et al. XPS and FTIR Studies of Polytetrafluoroethylene Thin Films Obtained by Physical Methods. *Polymers* **11**, 1629 (2019).
14. Li, J. et al. Multifunctional Artificial Artery from Direct 3D Printing with Built-In Ferroelectricity and Tissue-Matching Modulus for Real-Time Sensing and Occlusion Monitoring. *Adv. Funct. Mater.* **30**, 2002868 (2020).

15. Yuan, X. et al. A 3D-printed, alternatively tilt-polarized PVDF-TrFE polymer with enhanced piezoelectric effect for self-powered sensor application. *Nano Energy* **85**, 105985 (2021).
16. Chai, B. et al. Modulus-Modulated All-Organic Core-Shell Nanofiber with Remarkable Piezoelectricity for Energy Harvesting and Condition Monitoring. *Nano Lett.* **23**, 1810-1819 (2023).
17. Badatya, S., Bharti, D. K., Sathish, N., Srivastava, A. K. & Gupta, M. K. Humidity Sustainable Hydrophobic Poly(vinylidene fluoride)-Carbon Nanotubes Foam Based Piezoelectric Nanogenerator. *ACS Appl. Mater. Interfaces* **13**, 27245-27254 (2021).
18. Huang, X., Wang, Y. & Zhang, X. Ultrarobust, hierarchically anisotropic structured piezoelectric nanogenerators for self-powered sensing. *Nano Energy* **99**, 107379 (2022).
19. Shepelin, N. A. et al. Interfacial piezoelectric polarization locking in printable Ti3C2Tx MXene-fluoropolymer composites. *Nat. Commun.* **12**, 3171 (2021).
20. Tian, G. et al. Dielectric micro-capacitance for enhancing piezoelectricity via aligning MXene sheets in composites. *Cell Rep. Phys. Sci.* **3**, 100814 (2022).
21. Yang, T. et al. Hierarchically structured PVDF/ZnO core-shell nanofibers for self-powered physiological monitoring electronics. *Nano Energy* **72**, 104706 (2020).
22. Tian, G. et al. Ultrathin Epidermal P(VDF-TrFE) Piezoelectric Film for Wearable Electronics. *ACS Appl. Electron. Mater.* **5**, 1730-1737 (2023).
23. Deng, C. et al. Self-powered insole plantar pressure mapping system. *Adv. Funct. Mater.* **28**, 1801606 (2018).
24. Zhang, S., Zhang, B., Zhang, J. & Ren, K. Enhanced Piezoelectric Performance of Various Electrospun PVDF Nanofibers and Related Self-Powered Device Applications. *ACS Appl. Mater. Interfaces* **13**, 32242-32250 (2021).
25. Guo, W. et al. Wireless piezoelectric devices based on electrospun PVDF/BaTiO3 NW nanocomposite fibers for human motion monitoring. *Nanoscale* **10**, 17751-17760 (2018).
26. Bhavanasi, V., Kusuma, D. Y. & Lee, P. S. Polarization Orientation, Piezoelectricity, and Energy Harvesting Performance of Ferroelectric PVDF-TrFE Nanotubes Synthesized by Nanoconfinement. *Adv. Energy Mater.* **4**, 1400723 (2014).
27. Bhavanasi, V., Kumar, V., Parida, K., Wang, J. & Lee, P. S. Enhanced Piezoelectric Energy Harvesting Performance of Flexible PVDF-TrFE Bilayer Films with Graphene Oxide. *ACS Appl. Mater. Interfaces* **8**, 521-529 (2016).
