# Supplementary material for: All-polymer piezo-ionic-electric electronics
Source: Nat Commun. 2024 Dec 30;15:10876. doi: 10.1038/s41467-024-55177-y (PMC11686271; doi:10.1038/s41467-024-55177-y)
Supplement: Supplementary file 2 — Description of Additional Supplementary Files [file 41467_2024_55177_MOESM2_ESM.docx]

**Description of Additional Supplementary Files**

**File Name:** **Supplementary Video 1**

**Title: Thumb press PNP electronics to light up LED beads**

**Description:** A transducer application. The film portion of the PNP device (excluding the encapsulation layer) is only ~50 μm, and the one-sided Ag electrode is a circle with a radius of 6 mm. An acrylic plate is padded at the bottom for better stress on the film electronics. After circuit connection, the thumb press electronics is able to light up 26 LED beads in series, each with 5 mm specification and 1.8-2.0 V standard voltage.

**File Name:** **Supplementary Video 2**

**Title: PNP electronics is attached to the human wrist to accurately measure pulse waves**

**Description:** A sensor application. The flexible film electronics is attached to the wrist. by filtering out spurious waves, it is able to accurately measure the characteristic waves (P-waves, T-waves and D-waves) within each cycle of the pulse wave.

**File Name:** **Supplementary Video 3**

**Title: PNP electronics is attached to the human throat to roughly differentiate vocal cord vibration modes**

**Description:** A sensor application. The test subject uttered English words of different syllables, and the electronics attached to his throats was able to detect different waveforms. As the number of syllables in a word increases, the signal then becomes multi-peaked correspondingly.

**File Name:** **Supplementary Video 4**

**Title: PNP electronics is mounted on the tuning forks to detect frequency and amplitude**

**Description:** A sensor application. The film electronics is fixed to the head of 128 Hz or 256 Hz tuning fork. Knocking the tuning fork, the device generates voltage signals that increase in peak value with increasing impact force, reflecting changes in amplitude. After Fast Fourier Transform (FFT), the vibration frequency of the tuning fork (128 Hz or 256 Hz) is accurately displayed in the LabVIEW interface.
