# Supplementary material for: All-polymer piezo-ionic-electric electronics
Source: Nat Commun. 2024 Dec 30;15:10876. doi: 10.1038/s41467-024-55177-y (PMC11686271; doi:10.1038/s41467-024-55177-y)
Supplement: Supplementary file 7 — Reporting Summary [file 41467_2024_55177_MOESM7_ESM.pdf]

nature portfolio

Corresponding author(s): Prof. Weiqing Yang
Last updated by author(s): Nov 20, 2024

# Reporting Summary

Nature Portfolio wishes to improve the reproducibility of the work that we publish. This form provides structure for consistency and transparency in reporting. For further information on Nature Portfolio policies, see our Editorial Policies and the Editorial Policy Checklist.

## Statistics

For all statistical analyses, confirm that the following items are present in the figure legend, table legend, main text, or Methods section.

| n/a | Confirmed | |
|---|---|---|
| ☐ | ☒ | The exact sample size ($n$) for each experimental group/condition, given as a discrete number and unit of measurement |
| ☐ | ☒ | A statement on whether measurements were taken from distinct samples or whether the same sample was measured repeatedly |
| ☒ | ☐ | The statistical test(s) used AND whether they are one- or two-sided<br>*Only common tests should be described solely by name; describe more complex techniques in the Methods section.* |
| ☒ | ☐ | A description of all covariates tested |
| ☒ | ☐ | A description of any assumptions or corrections, such as tests of normality and adjustment for multiple comparisons |
| ☐ | ☒ | A full description of the statistical parameters including central tendency (e.g. means) or other basic estimates (e.g. regression coefficient) AND variation (e.g. standard deviation) or associated estimates of uncertainty (e.g. confidence intervals) |
| ☒ | ☐ | For null hypothesis testing, the test statistic (e.g. $F$, $t$, $r$) with confidence intervals, effect sizes, degrees of freedom and $P$ value noted<br>*Give P values as exact values whenever suitable.* |
| ☒ | ☐ | For Bayesian analysis, information on the choice of priors and Markov chain Monte Carlo settings |
| ☒ | ☐ | For hierarchical and complex designs, identification of the appropriate level for tests and full reporting of outcomes |
| ☒ | ☐ | Estimates of effect sizes (e.g. Cohen's $d$, Pearson's $r$), indicating how they were calculated |

*Our web collection on statistics for biologists contains articles on many of the points above.*

## Software and code

Policy information about availability of computer code

| | |
|---|---|
| Data collection | The cross-sectional morphology of the PNP was characterized using field emission scanning electron microscopy (FESEM, JSM-7800F, JEOL). The crystalline structure of PVDF was analyzed by X-ray diffraction (XRD, Empyrean, PANalytical). Fourier transform infrared (FTIR, Nicolet iS50, Thermo Fisher) spectroscopy was used characterize the PVDF compositional information. Polymer films were thermally analyzed using differential scanning calorimetry (DSC, Discovery DSC 2500, TA). The morphology result based on the atomic force microscope were obtained by the Multimode 8 (Bruker). Linear motors (LinMot H01-23×86/160) and force gauges (M7-50, Mark-10) constitute an excitation source system with real-time display of pressure values. All electrical signals were collected by the Data Acquisition Card (NI PCI-6221). |
| Data analysis | Data analysis for all curves was conducted utilizing the software packages Origin 2021.Potential simulations were executed by COMSOL Multiphysics 6.0.Molecular dynamics simulation in this work were performed by Materials Studio 2020 software. |

For manuscripts utilizing custom algorithms or software that are central to the research but not yet described in published literature, software must be made available to editors and reviewers. We strongly encourage code deposition in a community repository (e.g. GitHub). See the Nature Portfolio guidelines for submitting code & software for further information.

## Data

Policy information about availability of data

All manuscripts must include a data availability statement. This statement should provide the following information, where applicable:

- Accession codes, unique identifiers, or web links for publicly available datasets
- A description of any restrictions on data availability
- For clinical datasets or third party data, please ensure that the statement adheres to our policy

The data supporting the findings of this study are reported in the main text or the Supplementary Information. Raw data are provided as a Source Data file. All other data are available from the corresponding author upon request. Source data are provided with this paper.

## Research involving human participants, their data, or biological material

Policy information about studies with human participants or human data. See also policy information about sex, gender (identity/presentation), and sexual orientation and race, ethnicity and racism.

| | |
|---|---|
| Reporting on sex and gender | We recruited 2 volunteers (male). Gender was self-reported. The results were irrelevant to the gender of the participants. |
| Reporting on race, ethnicity, or other socially relevant groupings | The human participants in this study are students at Southwest Jiaotong University with the age from 20-30 years old. Population characteristics are not relevant to the experiment or the results of this study. |
| Population characteristics | 2 healthy subjects from 20-30 years old were involved in this study. |
| Recruitment | Participants were recruited from the School of Materials Science and Engineering at Southwest Jiaotong University. Participants were only required to be in good health. Self-selection bias, as well as other biases, will have no effect on the results of the experiment. |
| Ethics oversight | All testing from human participants was approved by the Institutional Review Boards of Southwest Jiaotong University with the reference number of SWJTU-23012-NSFC(133). Informed consent from all participants was obtained before inclusion in this study. |

Note that full information on the approval of the study protocol must also be provided in the manuscript.

# Field-specific reporting

Please select the one below that is the best fit for your research. If you are not sure, read the appropriate sections before making your selection.

☒ Life sciences ☐ Behavioural & social sciences ☐ Ecological, evolutionary & environmental sciences

For a reference copy of the document with all sections, see nature.com/documents/nr-reporting-summary-flat.pdf

# Life sciences study design

All studies must disclose on these points even when the disclosure is negative.

| | |
|---|---|
| Sample size | Only one participant needs to be selected for each experiment, as it is only needed to demonstrate the feasibility of the pulse wave detection experiment and the vocal cord vibration detection experiment. Comparisons between samples were not required for each experiment. The participant must simply be in good health. |
| Data exclusions | No data exclusions were involved in this research. |
| Replication | The results of the study are reproducible. All experiments were repeated more than 3 times to ensure accuracy. |
| Randomization | Only one participant per experimental group is required to verify feasibility, and it is sufficient that the participant is in good health. There is no need to consider sample randomization. |
| Blinding | As noted above, no group allocation is required. |

# Reporting for specific materials, systems and methods

We require information from authors about some types of materials, experimental systems and methods used in many studies. Here, indicate whether each material, system or method listed is relevant to your study. If you are not sure if a list item applies to your research, read the appropriate section before selecting a response.

## Materials & experimental systems

| n/a | Involved in the study |
|---|---|
| ☒ | ☐ Antibodies |
| ☒ | ☐ Eukaryotic cell lines |
| ☒ | ☐ Palaeontology and archaeology |
| ☒ | ☐ Animals and other organisms |
| ☒ | ☐ Clinical data |
| ☒ | ☐ Dual use research of concern |
| ☒ | ☐ Plants |

## Methods

| n/a | Involved in the study |
|---|---|
| ☒ | ☐ ChIP-seq |
| ☒ | ☐ Flow cytometry |
| ☒ | ☐ MRI-based neuroimaging |

# Plants

| | |
|---|---|
| Seed stocks | *Report on the source of all seed stocks or other plant material used. If applicable, state the seed stock centre and catalogue number. If plant specimens were collected from the field, describe the collection location, date and sampling procedures.* |
| Novel plant genotypes | *Describe the methods by which all novel plant genotypes were produced. This includes those generated by transgenic approaches, gene editing, chemical/radiation-based mutagenesis and hybridization. For transgenic lines, describe the transformation method, the number of independent lines analyzed and the generation upon which experiments were performed. For gene-edited lines, describe the editor used, the endogenous sequence targeted for editing, the targeting guide RNA sequence (if applicable) and how the editor was applied.* |
| Authentication | *Describe any authentication procedures for each seed stock used or novel genotype generated. Describe any experiments used to assess the effect of a mutation and, where applicable, how potential secondary effects (e.g. second site T-DNA insertions, mosiacism, off-target gene editing) were examined.* |
